# Comprehensive survey of condition-specific reproductive isolation reveals genetic incompatibility in yeast

**DOI:** 10.1038/ncomms8214

**Published:** 2015-05-26

**Authors:** Jing Hou, Anne Friedrich, Jean-Sebastien Gounot, Joseph Schacherer

**Affiliations:** 1 Department of Genetics, Genomics and Microbiology, University of Strasbourg/CNRS, UMR7156, 28 rue Goethe, 67083 Strasbourg, France

## Abstract

Genetic variation within a species could cause negative epistasis leading to reduced hybrid fitness and post-zygotic reproductive isolation. Recent studies in yeasts revealed chromosomal rearrangements as a major mechanism dampening intraspecific hybrid fertility on rich media. Here, by analysing a large number of *Saccharomyces cerevisiae* crosses on different culture conditions, we show environment-specific genetic incompatibility segregates readily within yeast and contributes to reproductive isolation. Over 24% (117 out of 481) of cases tested show potential epistasis, among which 6.7% (32 out of 481) are severe, with at least 20% of progeny loss on tested conditions. Based on the segregation patterns, we further characterize a two-locus Dobzhansky–Müller incompatibility case leading to offspring respiratory deficiency caused by nonsense mutation in a nuclear-encoding mitochondrial gene and tRNA suppressor. We provide evidence that this precise configuration could be adaptive in fluctuating environments, highlighting the role of ecological selection in the onset of genetic incompatibility and reproductive isolation in yeast.

## Abstract (web summary)

Chromosomal rearrangements may hamper intraspecific hybrid fertility. Here the authors show that environment-specific genetic incompatibility segregates readily within intermating populations and leads to intrinsic reproductive isolation within a yeast species.

## Graphical abstract

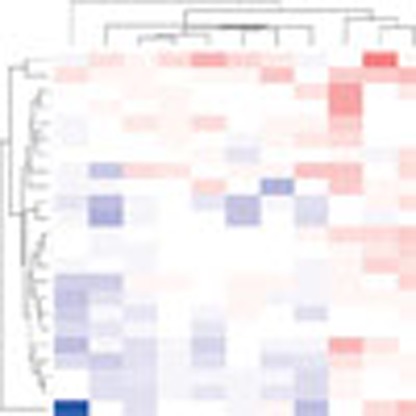

Genetic variation accumulated in different natural populations could occasionally cause deleterious epistatic effects leading to reduced hybrid fitness and intrinsic post-zygotic reproductive isolation. The Dobzhansky–Müller model described such negative epistasis as genetic incompatibility, where independently fixed mutations in allopatric populations could not properly function together when combined in hybrids^1^. Although most prominently studied between closely related species^2^,^3^,^4^,^5^,^6^,^7^,^8^,^9^, recent efforts have been made to characterize genetic incompatibilities within the same species, and it has been shown that deleterious epistasis segregates readily at an intraspecific scale^10^,^11^,^12^,^13^,^14^,^15^,^16^. The current focus on intraspecific genetic incompatibilities underscores the importance of the ongoing phenotypic consequences of genetic diversity within an inter-mating population, and captures the evolutionary origin of the early onset of reproductive isolation and speciation.

In different model organisms, multiple molecular mechanisms have been identified to explain the observed genetic incompatibility within populations. For example, in *Caenorhabditis elegans*, the specific allelic combination of a paternal-delivered toxin *peel-1* and a zygotic-acting antidote *zeel-1* was incompatible, which led to F2 offspring inviability in a cross between Hawaii and Bristol isolates^10^,^17^. Another example in *Arabidopsis thaliana* emphasized the role of genetic drift, where reciprocal inactivation of a duplicated essential gene histidinol-phosphate aminotransferase (*HPA*) in different parental genomes led to F2 segregation distortion^12^. In addition, several studies highlighted the effect of the molecular arms race for genes involved in the plant pathogen defence immune system leading to hybrid necrosis^18^,^19^,^20^. More recently, a systematic analysis across the species *A. thaliana* further confirmed the essential role of plant autoimmunity and identified several hot spots leading to hybrid necrosis^16^. However, regardless of the molecular causes, the evolutionary origin of incompatibilities depends largely on the life history context of a species. The interplay between selection and drift could play a prominent role in shaping genetic variation and their phenotypic outcome in nature. Systematic exploration of intraspecific reproductive isolation in additional species is therefore valuable to obtain a global picture of the evolutionary and molecular mechanisms that could be involved in various species.

Yeasts are free-living unicellular eukaryotes, which can be isolated from various ecological (for example, tree exudate, wine, different fermentations and immuno-compromised patients) and geographical (Europe, Asia, Africa and America) niches^21^. Genetic variation accumulated over their evolution in these niches constitutes a rich repertoire of potential variants leading to reproductive isolation. Recent efforts of species-wide surveys of post-zygotic reproductive isolation within yeasts showed frequent occurrence of reduced hybrid fertility when crossing genetically divergent parental isolates, where the observed cases were shown to be mostly due to the presence of large-scale chromosomal rearrangements that led to the unbalanced distribution of gene sets in the offspring^14^,^15^. By contrast, no evident case of classic genetic incompatibility has been found so far in natural populations of yeast. Nevertheless, as most studies were only focused on offspring viabilities in permissive laboratory conditions, that is, rich media yeast extract peptone dextrose (YPD) at 30 °C, the extent to which different environmental conditions has a part in the onset of reproductive isolation was largely overlooked.

Here, we perform a global evaluation of the onset of genetic incompatibility across various environments using the yeast *Saccharomyces cerevisiae*. We measure the offspring viability of 27 crosses on a large number of conditions (different carbon sources, chemicals and temperatures). We show condition-specific incompatibilities are frequent: over one-fourth of the tested cases show loss of offspring viability to varied degrees (from 1 to 62%), possibly indicating the presence of negative epistasis. We analyse the segregation patterns of identified cases and focus on one case that demonstrated a potential recessive two loci Dobzhansky–Müller incompatibility related to the loss of offspring viability on media containing non-fermentable carbon sources (for example, glycerol and ethanol). By further analysing the genes and mutations involved, we show that the incompatibility is due to the presence of a nonsense mutation in a nuclear-encoding mitochondrial gene and a tRNA nonsense suppressor. This is the first identified case of classic two loci Dobzhansky–Müller genetic incompatibility within a yeast species. To identify the evolutionary origin of the onset and maintenance of this particular configuration, we evaluate the effect of the suppressor in different genetic backgrounds and show that the presence of this suppressor confers a surprising level of phenotypic variation, which could potentially offer selective advantages across various environmental stresses. Interestingly, despite frequent observation of tRNA suppressors in laboratory selections^22^,^23^,^24^,^25^,^26^, the occurrence of such suppressors in natural isolates of yeast is extremely rare, possibly suggesting a transient role of suppressors in adaptation in *S. cerevisiae*.

## Results

### Survey of environment-dependent reproductive isolation

**Figure 1:**
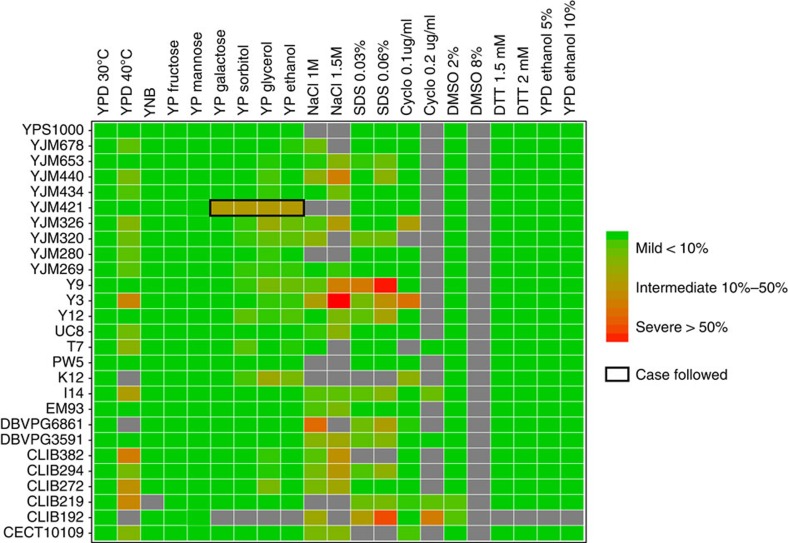
Offspring viability of 27 natural isolates crossed with S288c on 20 conditions. Offspring viabilities estimated based on 20 full tetrads are colour coded with the vertical axis representing isolates crossed and horizontal axis representing the 20 conditions tested. All isolates were previously shown to produce high offspring viability (>90%) on YPD when crossed with S288c^15^. Conditions where either one or both parental strains were non-viable are coloured in grey. The case followed is circled in black.

In total, 27 natural isolates previously shown to be compatible with the reference strain S288c (offspring viability >90% on YPD) were selected^15^. All isolates were crossed with S288c and offspring viability was scored and confirmed on YPD (Supplementary Table 1). For each cross, 20 full tetrads (containing only viable spores) were chosen to be tested on 20 conditions, including different temperatures, carbon sources and various chemical compounds (Fig. 1 and Supplementary Table 2). This summed up to a total of 540 instances spanning 27 crosses on 20 conditions (Fig. 1 and Supplementary Fig. 1). Among all 540 instances assessed, 59 involved at least one parental strain being non-viable on the condition tested and were excluded for further analysis (Fig. 1). Overall, 24.3% of all instances (117 out of 481) showed signs of negative epistasis with different degrees of loss of offspring viability ranging from 1 to 62% (Fig. 1). Among these cases, 6.7% (32 out of 481) showed moderate to severe incompatibility, with at least 20% of the segregants being non-viable on the condition tested (Fig. 1).

### Potential case of two loci Dobzhansky–Müller incompatibility

**Figure 2:**
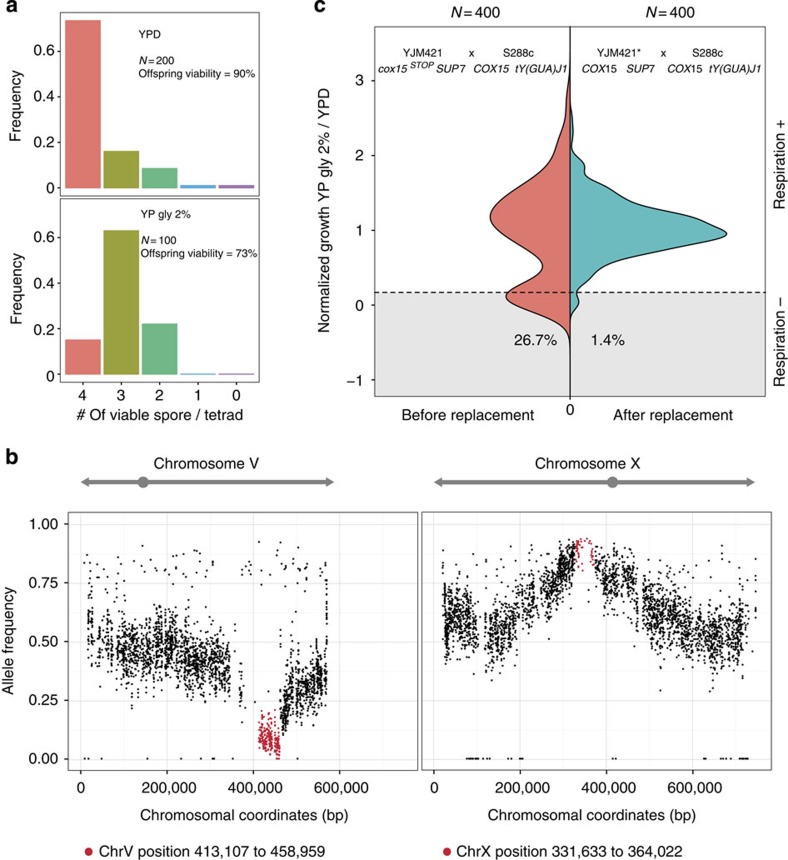
Molecular characterization of the incompatible case related to respiratory deficiency between YJM421 and S288c. (**a**) Phenotypic segregation of the cross. The frequency of tetrads containing 4, 3, 2, 1 or 0 viable segregants was presented for YPD (upper plot) and YP glycerol 2% (lower plot). The number of tetrads tested is as indicated. (**b**) Genomic regions with skewed allele frequencies identified using bulk segregant analysis. Eighty non-viable segregants on 2% YP glycerol were pooled and sequenced and two candidate regions were identified. The horizontal axis represents the coordinates of chromosome V and X. The vertical axis corresponds to the allele frequencies of S288c. Identified regions are coloured in red. (**c**) Comparison of the phenotype distributions of the segregants obtained before and after allele replacement of *COX15*. The normalized growth ratio of 400 segregants from the cross between YJM421 (*cox15*^*stop*^*/SUP7*; left panel) or YJM421* (*COX15/SUP7*; right panel) and S288c are presented as colour coded frequency distributions. Shaded areas indicate the fractions of segregants that are respiratory deficient.

To assess the genetic complexity of the observed cases, we analysed the segregation patterns of the lethal phenotype (Supplementary Fig. 1). Most of the cases were consistent with complex epistasis and were not characterized in this study (∼103 out of 117, Supplementary Fig. 1). We focused on one cross between a clinical isolate YJM421 (ref. 27) and S288c, which showed a clear pattern of recessive two loci Dobzhansky–Müller incompatibility in several conditions related to respiration efficiency (2% YP sorbitol, 2% YP glycerol, 2% YP ethanol and 2% YP galactose). In this scenario, the lethal allelic combination should follow Mendelian segregation, which leads to one-fourth in the loss of viability in the offspring, resulting in a ratio of 1:4:1 for tetrads containing 4, 3 or 2 viable spores, respectively, assuming the interacting loci are independent. For this cross, additional tetrads were tested on 2% YP glycerol and the segregation pattern was confirmed (Fig. 2a). Approximately 25% of the offspring were respiration deficient and were unable to grow on 2% YP glycerol.

### Mapping of the loci involved using bulk segregant analysis

To map the loci involved, we used a bulk segregant analysis strategy followed by whole-genome sequencing (BSA-seq)^15^. Briefly, 80 segregants that were non-viable on 2% YP glycerol from independent tetrads were pooled and sequenced. The sequences obtained were aligned to the genome of S288c and the allele frequency of S288c was scored at each polymorphic position. For most genomic regions, the expected allele frequency for both parental strains was ∼0.5, whereas the loci involved in the incompatibility would have deviated allele frequencies. Using this strategy, we mapped two regions with significant allele frequency deviation (see details in Methods), one located on the right arm of chromosome V (position 413,107 to 458,959) and the second on the left arm of chromosome X (position 331,633 to 364,022), spanning approximately 46 and 33 kb, respectively (Fig. 2b).

### Identification and functional validation of candidate genes

To identify the causative genes for the observed respiratory deficiency, we closely examined the mapped regions for potential candidates involved in respiration. In total, five genes in these regions were potentially involved in respiration according to the SGD annotations ( http://www.yeastgenome.org/), among which three were found in the region on chromosome V (*EMP65*, *COX15* and *FTR1*) and two in the region on chromosome X (*TIM54* and *AIM22*). We examined the DNA sequences of these genes in the YJM421 background and found a nonsense mutation at the position +115 in the open reading frame of *COX15* (CAA to TAA; position 453574 on chromosome V). *COX15* encodes an inner membrane cargo protein in the mitochondria, the function of which is essential for respiration^28^. The observation of a nonsense mutation in this gene was surprising as the presence of such mutation would likely abolish the function of *COX15* and lead to respiratory deficiency; whereas the strain YJM421 carrying the mutation was respiratory competent.

To verify if the YJM421 allele of *COX15* (*cox15*^*stop*^) was functional, we deleted the *cox15*^*stop*^ in YJM421, and the resulting mutant was unable to grow on media containing non-fermentable carbon sources (Supplementary Fig. 2a). Moreover, allele replacement of *cox15*^*stop*^ with the wild-type *COX15* from S288c in the YJM421 background resulted in total rescue of the genetic incompatibility observed: cross between YJM421 *cox15*^*stop*^*::COX15* and S288c led to 98.6% offspring viability on YP glycerol 2% (400 segregants tested; Fig. 2c). These results confirmed that *cox15*^*stop*^ was functional in the YJM421 background and was involved in the incompatibility between YJM421 and S288c. The fact that *cox15*^*stop*^ was functionally active strongly suggests the presence of a genetic element at the interacting loci on chromosome X that compensates the effect of the nonsense mutation in YJM421.

Indeed, when examining the DNA sequence of YJM421 in the mapped region on chromosome X, we found a mutation at the anticodon position of a tyrosine tRNA tY(GUA)J1 (GTA to TTA, position 354280 on chromosome X), which in turn transformed this tRNA into a TAA nonsense suppressor (*SUP7*). The presence of this suppressor would effectively read-through the premature stop codon in *cox15*^*stop*^, which leads to a functional protein product in YJM421. However, this configuration of *cox15*^*stop*^/*SUP7* in YJM421 renders the strain incompatible when crossed with S288c, as one-fourth of the segregants would inherent only the non-functional *cox15*^*stop*^ allele but not the suppressor, leading to respiratory deficiency. To confirm this hypothesis, we transformed segregants that are non-viable on 2% YP glycerol with a yeast centromeric plasmid containing the suppressor *SUP7* (CEN_SUP7), and confirmed that the presence of this suppressor restored their respiration capacity (Supplementary Fig. 2b). These results demonstrated the first identified pair of Dobzhansky–Müller incompatibility genes within a yeast species. Nevertheless, the evolutionary and physiological implications of this specific combination of *cox15*^*stop*^/*SUP7* are still unclear.

### Differential fitness effect of *SUP7* in diverse isolates

**Figure 3:**
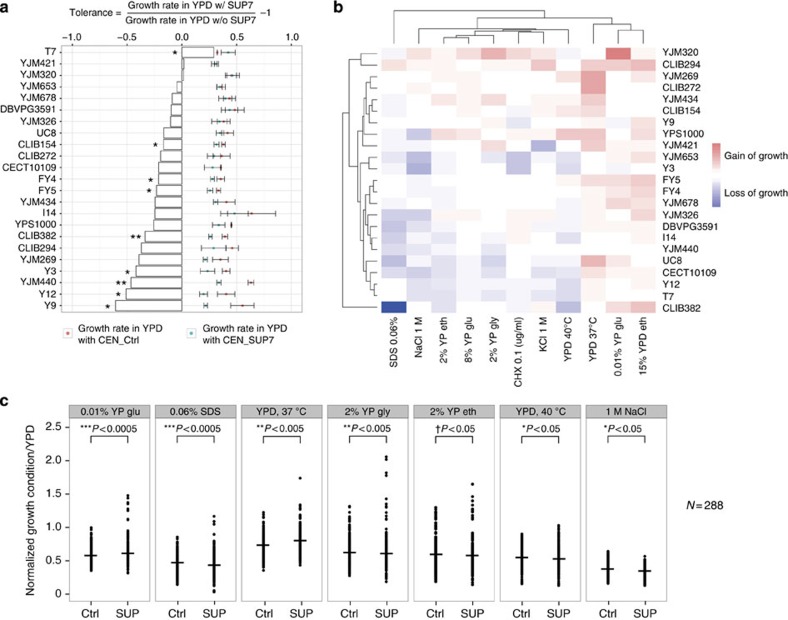
Phenotypic consequences of *SUP7* in various isolates. (**a**) Growth variation in the presence of *SUP7* in non-stressful conditions (YPD, 30 °C). Growth rates of 23 strains measured in liquid YPD at 30 °C in the presence of a plasmid containing *SUP7* (blue dots) or empty control vector (red dots) are presented with error bars (mean±s.d.; *N*=6). Percentage of growth variation is calculated and presented as bars (*N*=6; two-sided *t*-test *P value<0.05; **P value<0.005). (**b**) Suppressor induced phenotypic variation in stress conditions. Significant variation of the normalized growth ratio (>10%) due to the presence of *SUP7* of 23 strains is presented for 11 stress conditions using a heatmap with blue indicating loss of growth and red for gain of growth compared with strains carrying the empty control vector. (**c**) Significant increase of phenotypic variance in the presence of *SUP7.* Distribution of the normalized growth ratio in stress conditions was compared for strains carrying the suppressor *SUP7* (SUP) or control (Ctrl) in seven conditions. Median values for each condition are indicated with a bar. Statistical significance is as shown on the plot (*N*=288, two-sided F-test *P value<0.05, **P value <0.005, ***P value<0.0005; Levene test ^†^*P* value<0.05).

In fact, tRNA suppressors are well known to effectively suppress nonsense mutations by stop codon read-through, although the presence of such suppressors is likely detrimental because of the perturbation of cellular translational fidelity. As the incompatible strain YJM421 did not show any apparent growth defect, we sought to evaluate the effect of the suppressor *SUP7* on growth in different genetic backgrounds. We transformed 23 diverse natural isolates with a plasmid containing *SUP7* (CEN_SUP7) or an empty control plasmid (CEN_Ctrl) and measured their growth rate in a non-stressful condition (YPD at 30 °C) using microcultures (Supplementary Table 1). A mean reduction of the growth rate across all strains tested was observed (mean reduction 22.8%, *N*=138, two-sided *t*-test *P-*value <<0.005), with the most severe case of 2.53-folds lower growth in the presence of the suppressor compared with the strain carrying the control plasmid (Fig. 3a; strain Y9 with two-sided *t*-test *P-*value<0.05, *N*=6). Interestingly, despite an overall deleterious effect of *SUP7*, several isolates, including YJM421, YJM320 and T7, showed similar or higher growth rates in the presence of the suppressor (Fig. 3a). These results suggest that the effect of *SUP7* on growth is background dependent and different levels of genetic assimilation could be observed, such as the case for YJM421, thus allowing for the persistence of *SUP7* in this strain.

### Impacts of *SUP7* in stress conditions across natural isolates

To further investigate the phenotypic consequences of *SUP7*, we evaluated the fitness of the same set of 23 isolates carrying the plasmid with *SUP7* (CEN_SUP7) on solid media for various stress conditions (membrane stability, proteome perturbation, osmotic stress, different carbon sources and high temperatures; Fig. 3b and Supplementary Table 2). The normalized growth ratio was calculated by comparing the colony size on tested conditions versus YPD to eliminate the effects of growth differences on YPD and pinning density on solid plates. We then calculated the percentage of fitness variation for each isolate in the presence of *SUP7* compared with the same isolate carrying the control plasmid on each condition. Significant variation due to the presence of *SUP7* was observed in most of the conditions tested, with nearly half of the cases showing a gain of fitness higher than 10% (Fig. 3b). These variations appeared to be strain and condition specific, with exceptions for some conditions (YPD 37 °C and YPD ethanol 15%) where all strains grew better in the presence of *SUP7*, and some strains (CLIB294, YJM269 and CLIB272) with an overall gain of fitness across all conditions. In addition, for most of the conditions tested (7 out of 11), significantly increased phenotypic variance was observed in the presence of *SUP7* compared with the controls across all strains (Fig. 3c and Supplementary Fig. 3). These results suggest that the suppressor *SUP7* contributes to marked phenotypic variation across different genetic backgrounds in stress conditions, and carrying the suppressor might, in turn, offer some selective advantages in the presence of environmental challenges.

### Frequency of nonsense mutation and tRNA suppressor in yeast

**Figure 4:**
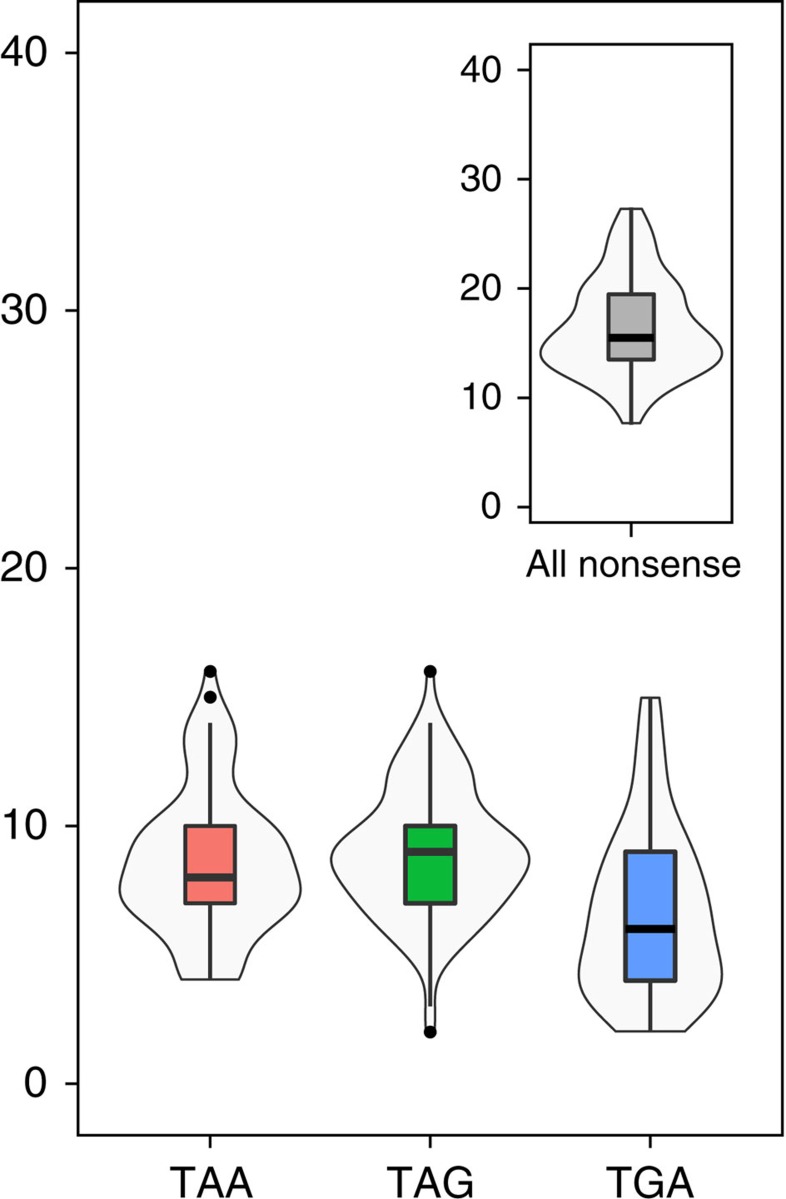
Nonsense mutations in natural populations. Distribution and number of nonsense mutations in verified ORFs across 100 sequenced natural isolates^29^,^30^. Mutations in different stop codon classes are colour coded.

To explore the prevalence of nonsense mutations and tRNA suppressors in natural populations of yeast, we surveyed 100 genomes of *S. cerevisiae* that are publically available^29^,^30^. Compared with common lab strains, nonsense mutations in natural isolates were quite frequent, with an average of ∼10 nonsense mutations in each stop codon class per strain (Fig. 4). The frequency of nonsense mutations globally followed a normal distribution, with a maximum frequency of nonsense mutations per strain of ∼16 mutations for each anticodon class (Fig. 4). Genes bearing any class of nonsense mutations were functionally enriched for stress-related activities, such as transmembrane transporter activity, detoxification and transcription regulation (Supplementary Table 3). More than 40% (215 out of 500) of the detected genes with nonsense mutations were shared by at least two isolates.

In addition to nonsense mutations, we also looked for the presence of potential tRNA suppressors in these genomes. No tRNA suppressor of any anticodon class was found in this rather large data set. In contrast to the prevalence of nonsense mutations in natural populations, the frequency of tRNA suppressors is extremely rare, possibly suggesting a transient role of the suppressors in adaptation in *S. cerevisiae*.

## Discussion

We performed a species-wide survey of environment-dependent reproductive isolation and identified the first Dobzhansky–Müller incompatibility gene pair related to offspring respiratory deficiency within the yeast species *Saccharomyces cerevisiae*. We showed the incompatibility was due to a combination of a nonsense mutation in *COX15* and a tRNA suppressor *SUP7* in a single isolate, which leads to one-fourth of the offspring having only a non-functional copy of *COX15* upon crossing with the reference strain S288c. We also provided evidence that the persistence of this particular allelic combination might potentially be related to increased evolutionary potential when facing fluctuating environmental conditions in nature. Our study highlights the importance of understanding the ecological context to hybrid fitness and extends the overview of possible mechanisms involved in the onset of intraspecific post-zygotic reproductive isolation in yeast.

In yeasts, multiple mechanisms, such as chromosomal rearrangements, anti-recombination, cyto-nuclear incompatibility and meiotic drive elements, have been identified to explain the observed loss of hybrid fertility between different species^7^,^8^,^31^,^32^,^33^,^34^,^35^. However, the relative role of Dobzhansky–Müller genetic incompatibility to the onset of reproductive isolation in yeasts has long been a subject of debate, primarily due to lack of empirical support^6^,^36^,^37^,^38^. At the intraspecific level, large-scale chromosomal rearrangements such as reciprocal translocations were considered to be the major mechanism leading to reduced offspring viability when crossing natural populations^14^,^15^, whereas cases of deleterious genic interactions were found to be rare, with the only example demonstrated in *S. cerevisiae* related to interactions between genes in the mismatch repair system leading to sporadic progeny loss^39^,^40^. In addition to these mechanisms, here we showed that classic two loci Dobzhansky–Müller incompatibilities do exist in natural isolates of yeast and could readily lead to reproductive isolation in different environmental conditions. Although the conditions investigated in the current study do not represent the true ecological contexts encountered, it is evident that the overall picture of molecular mechanisms affecting reproductive traits in nature is far more complex than previously envisioned within a yeast species.

Nevertheless, the evolutionary forces driving the onset and maintenance of isolating mechanisms are still in question. In the identified case of respiration-related genetic incompatibility, both mutations were found in the genome of the incompatible strain YJM421, resulting in a ‘derived-ancestral’ type of interaction. This observation confirmed that the onset of genetic incompatibility does not necessarily require independent fixation of causative mutations in allopatry, as was initially proposed by the Dobzhansky–Müller model^1^. However, although the origin of the onset and maintenance of this particular configuration is unclear, several possibilities could be envisioned. On one hand, as the YJM421 strain showed phenotypic tolerance of the suppressor *SUP7* (Fig. 3a), it is possible that this suppressor was acquired in conditions where possessing a suppressor was beneficial. In this scenario, the loss-of-function allele of *cox15*^*stop*^ might arise due to random genetic drift, the effect of which was buffered by the pre-existence of *SUP7.* Alternatively, the fixation of the *cox15*^*stop*^ could arise before the apparition of *SUP7*. In fact, the incompatible isolate YJM421 was of clinical origin and it has been shown that deletion of *COX15* could confer higher levels of resistance to antifungal drugs and biofilm formation, two traits that are particularly advantageous for clinical propagation^41^. In this scenario, it is possible that the loss-of-function allele *cox15*^*stop*^ originally arose due to selection pressure in clinical conditions. When the strain was replaced in favourable conditions and the original selective pressure was removed, the suppressor could arise to rescue the loss of respiratory capacity because of the adaptation to a new environment. This strain could then become integrated in subsequent genetic and phenotypic assimilation, allowing the particular allelic combination to persist. Even though these scenarios remain only conjectures, it is likely that environmental fluctuation and selection might at least partly contribute to the onset and maintenance of this particular case.

tRNA suppressors perturb the translational fidelity by stop codon read-through, the effect of which resembles the yeast prion [PSI+]^42^,^43^. In *S. cerevisiae*, tRNA suppressors have been frequently selected in numerous genetic screens^22^,^25^,^26^. However, how frequently such suppressors occur in natural isolates was unknown. We surveyed over 100 publically available genomes of *S. cerevisiae* natural isolates and found no tRNA suppressor of any known family in these genomes except for that of YJM421. The scarcity of tRNA suppressors in natural isolates in contrast with their relatively high frequency of occurrence in the presence of strong selection suggests that tRNA suppressors might represent a transient survival mechanism, which could subsequently be lost in the absence of selection^44^. Nevertheless, the evolutionary fate of such suppressors probably depends on the specific genomic and environmental context of the strain in question. In fact, we demonstrated that the presence of the suppressor *SUP7* conferred an increased phenotypic capacity across multiple stress conditions, possibly fuelled by the relatively high number of naturally occurring nonsense mutations in natural isolates. Therefore, much like the prion [PSI+]^45^, tRNA suppressors could offer context-dependent selective advantages in yeast. However, as opposed to prions, tRNA suppressors are stably transmitted in a Mendelian manner, which in turn could drive the fixation of allelic combinations leading to the onset of negative epistasis, as is evident in the identified case of genetic incompatibility.

By taking into account environmental factors in the onset of reproductive isolation across a large number of crosses in *S. cerevisiae*, we revealed that context-dependent negative epistasis readily segregates in this species and the frequency of which might be more common than previously thought. Nevertheless, cases of the classic two loci Dobzhansky–Müller incompatibility appeared to be rare, with most cases of identified potential negative epistasis apparently reaching a higher complexity, even at an intraspecific scale. The origin of such epistasis was potentially due to the combinatory effect of selection and drift. Further understanding of the onset of intraspecific genetic incompatibilities will extend our perspectives regarding the ongoing phenotypic consequences of genetic diversity within a species, as well as the underlying evolutionary forces that shape the patterns of such variation.

## Methods

### Strains

A collection of 27 strains isolated from diverse ecological (tree exudate, wine, different fermentations and clinical) and geographical (Europe, Asia, Africa and America) origins was used in this study for crossing, phenotyping and survey of nonsense mutations (Supplementary Table 1). Laboratory strains isogenic to S288c, FY4 (*MAT*a) and FY5 (*MAT*α) were also used. A uracil auxotrophic mutant of YJM421 (YJM421 *ura3Δ0*) was generated by deleting the *URA3* gene using 5-FOA selection.

### Media and culture conditions

Standard media conditions were used (YPD 1% yeast extract, 2% peptone and 2% glucose at 30 °C) for growth and maintenance of the strains. A final concentration of 200 μg ml^−1^ hygromycin (Euromedex) was supplemented to maintain the plasmid CEN_SUP7 or CEN_Ctrl carrying a resistance marker gene hygroMX. Sporulation was induced on potassium acetate plates (1% potassium acetate, 2% agar). The 5-FOA selection plate was made by supplementing 25 μg ml^−1^ uracil and 1 mg ml^−1^ 5-FOA in a synthetic complete uracil dropout media (SC-URA). Detailed media composition for the screening of offspring viability and stress conditions are listed in Supplementary Table 2. All procedures are performed at 30 °C unless otherwise indicated.

### Screen of offspring viability on various conditions

The 27 strains are all *MAT*α and were previously shown to be compatible when crossed with FY4 (*MAT*a, isogenic to S288c)^15^. High offspring viability (>90%) of all crosses was confirmed using microdissection on YPD using a micromanipulator (Singer MSM-400) after asci digestion by zymolyase (MT ImmunO 20 T). For each cross, 20 full tetrads (containing 4 viable spores) were suspended in liquid YPD and pinned onto corresponding condition plates as well as a YPD control plate using a frogger. Growth was scored by eye after 48 h where viable segregants formed a patch. The offspring viability for each cross and condition corresponds to the ratio between the number of viable segregants and the total number of segregants viable on YPD. See Supplementary Table 1 and Supplementary Fig. 1 for detailed offspring viability and phenotype segregation for each tested case.

### Bulk segregant analysis strategy

For the cross between YJM421 and S288c, 25% of the segregants were respiratory deficient and not viable on 2% YP glycerol. In total, 80 independent respiratory-deficient segregants were separately cultured then pooled by equal OD readings at 600 nm and the DNA was sequenced. Causative genes for the observed incompatibility are presumably united in these segregants and genomic regions involved are mapped by analysing the allele frequency variation.

### DNA extraction, sequencing, SNP calling and data analysis

Genomic DNA was extracted using the Qiagen Genomic-tip kit. DNA of the pooled segregants was sequenced using Illumina Hiseq 2000. Detailed procedures of DNA preparation were described previously^46^. We used paired-end libraries, 101 bp per read, the coverage was ∼50 × . Reads were aligned to the S288c genome using BWA with ‘-n 5 -o 2’ options. Single-nucleotide polymorphism (SNP) calling was done using SAMtools^47^. The allele frequency of S288c was scored at each polymorphic position. As indicated by the segregation pattern, two genomic regions enriched for each parental strain are expected. We defined the region involved in the YJM421 parent as the genomic region comprising most of the mapped SNPs with allele frequency of S288c <0.1, as the S288c alleles were supposed to be absent (chromosome V). As for the region involved in the S288c genome, the YJM421 alleles should have been absent, which would result in a region with no mapped SNP markers (chromosome X). Inconsecutive SNPs that showed high allelic enrichment are considered to be sequencing noise and were not considered in the analysis.

### Plasmid construction and transformation

Empty centromeric Gateway plasmid with hygroMX as a resistance marker was kindly provided by Dr S. Treusch (CEN_Ctrl)^48^. The fragment containing *SUP7* was amplified with its original regulatory regions from the genomic DNA of YJM421, tagged with attB1/attB2 sites compatible with the Gateway cloning system (Invitrogen). Cloning of the fragment was performed according to instruction. The resulting plasmid (CEN_SUP7) was verified using restriction enzymes as well as Sanger sequencing. Transformation of yeast strains was performed using EZ transformation kit (MP biomedicals). Single colonies of transformants were maintained on YPD media containing 200 μg ml^−1^ hygromycin.

### Growth measurements using microculture

To measure the growth of strains in the presence or absence of the suppressor in non-stressful condition, strains tranformed with the corresponding plasmids (CEN_Ctrl or CEN_SUP) were pregrown for 24 h in YPD containing 200 μg ml^−1^ hygromycin and then transferred into 150 μl of the same media in flat bottom 96-well plates (Nuclon, ThermoFischer) using a long pin replicator. Cultures were grown in six replicates for 48 h and the absorbance of each well was read at 595 nm in 10 min intervals (four spatial positions and three flashes) using a TECAN plate reader (Infinite series) with horizontal and orbital shaking. Growth curves were retrieved using the programme GATHODE^49^ and the growth rate was calculated using an exponential curve fit.

### Quantitative phenotyping using solid media

To measure growth variation due to the presence of *SUP7* in stress conditions, 23 isolates carrying different plasmids (CEN_Ctrl or CEN_SUP) were pregrown in liquid YPD with 200 μg ml^−1^ hygromycin and pinned onto a solid YPD plate with hygromycin to a 384 format using a replicating robot RoToR (Singer instruments). The resulting matrix plate was allowed to grow for 48 h at 30 °C, and was subsequently replicated on 12 different conditions including YPD as a pinning control. All media conditions tested were supplemented with 200 μg ml^−1^ hygromycin to maintain the plasmids, detailed compositions are listed in Supplementary Table 2. Plates were scanned for colony size at three time points (24, 40 and 48 h) and analysed using the R package Gitter^50^. Each strain was tested in 12 replicates and growth was measured by analysing colony size. Normalized growth ratios were calculated first by normalizing the colony size by the growth on YPD for pinning effect, then by the value of the strain carrying the suppressor (CEN_SUP) as a control (CEN_Ctrl) for each condition. All calculations and statistical analyses were performed using R.

### Annotation of nonsense mutations and functional enrichment

A total of 100 recently sequenced isolates of *S. cerevisiae* (NCBI BioProject PRJNA189847 to PRJNA189936, PRJNA189300, PRJNA188959, PRJEB2299 and refs 29, 30) were used for nonsense mutation detection. The reads were retrieved and cleaned using cutAdapt, and aligned to the reference genome S288c. Read alignments and SNP calling were performed as before and SNPs were annotated using the EMBL annotation using a customized Python script. Gene Ontology (GO) term enrichment was performed using FunSpec^51^.

### Genome assemblies and detection of tRNA suppressor

For the same set of 100 strains, cleaned reads were assembled using SOAPdenovo2 (ref. 52), version 2.04, with a k-mer size of 75. The assemblies were then surveyed for potential tRNA suppressors using tRNAscan-SE (version 1.3.1) and no suppressor of any codon families were found in this data set.

## Additional information

**Accession codes**: The short-read sequences of the pool of segregants generated in this study have been deposited in the NCBI Sequence Read Archive (SRA) database under the accession codes PRJNA280047 and SRP056771.

**How to cite this article**: Hou, J. *et al.* Comprehensive survey of condition-specific reproductive isolation reveals genetic incompatibility in yeast. *Nat. Commun.* 6:7214 doi: 10.1038/ncomms8214 (2015).

## Supplementary Material

Supplementary Information Supplementary Figures 1-3, Supplementary Tables 1-3 and Supplementary Reference
