# Supplementary material for: Comprehensive survey of condition-specific reproductive isolation reveals genetic incompatibility in yeast
Source: Nat Commun. 2015 May 26;6:7214. doi: 10.1038/ncomms8214 (PMC4445460; doi:10.1038/ncomms8214)
Supplement: Supplementary Information — Supplementary Figures 1-3, Supplementary Tables 1-3 and Supplementary Reference [file ncomms8214-s1.pdf]

Supplementary Figure 1.

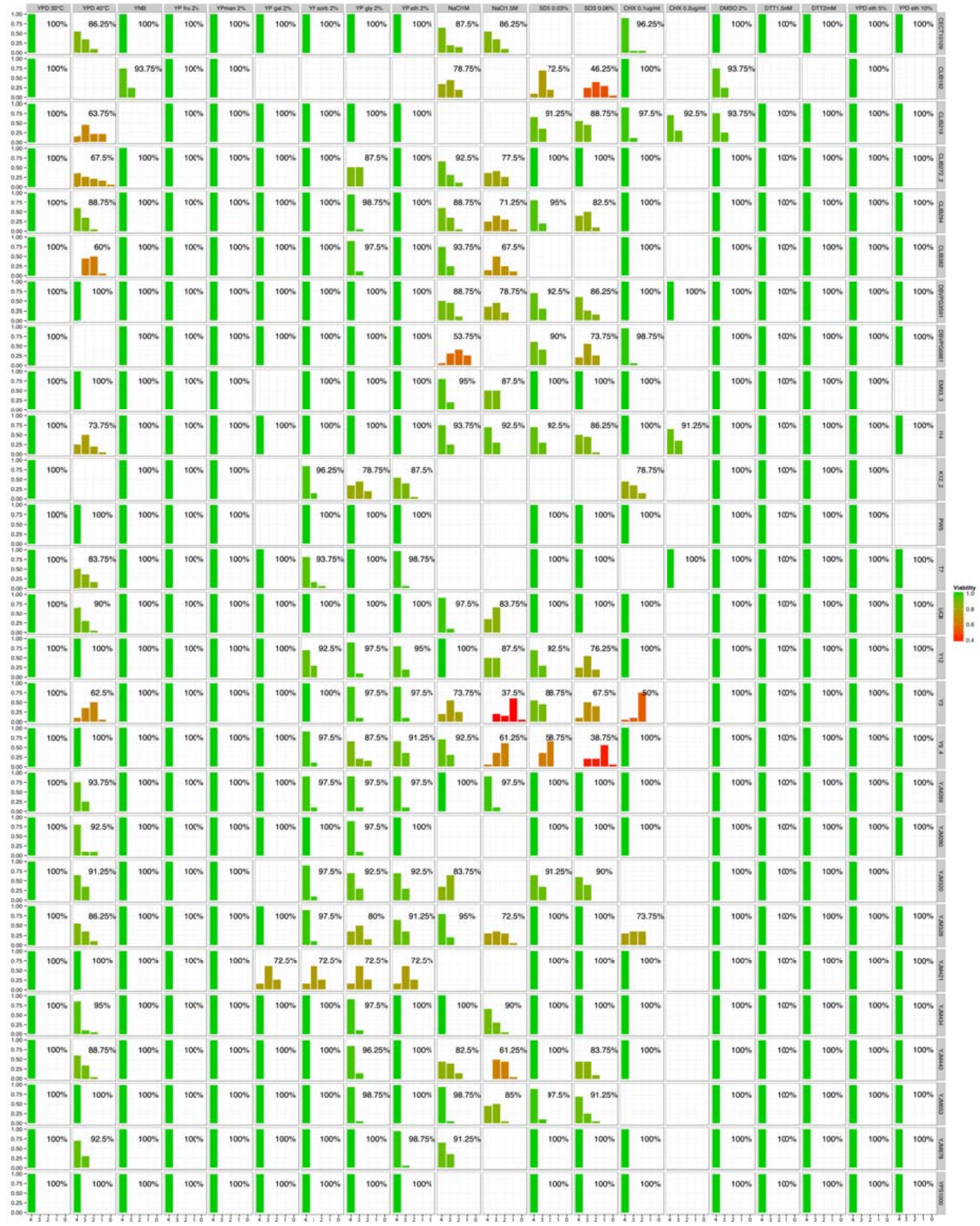

Supplementary Figure 1, related to Figure 1 - Lethal phenotype segregation of 27 crosses on 20 conditions. Segregation of 20 full tetrads (containing 4 viable spores on YPD) tested for each cross (vertical axis) and each condition (horizontal axis) are presented. The resulting offspring viabilities are colour coded.

Supplementary Figure 2.

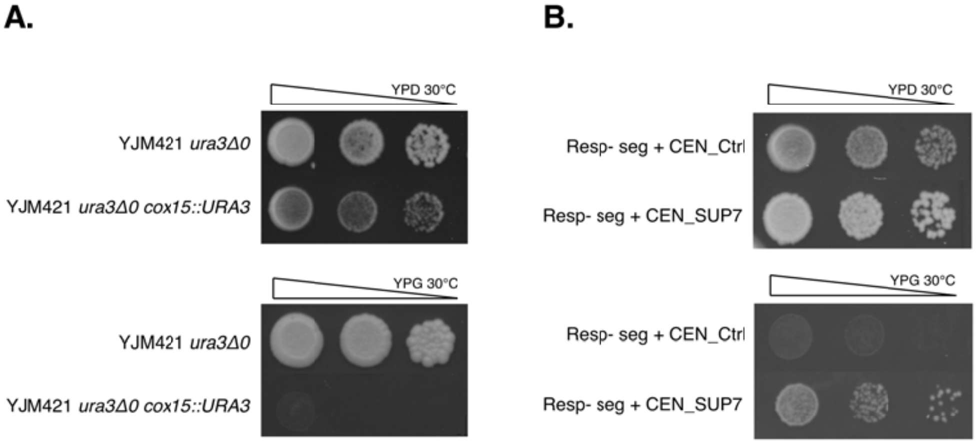

Supplementary Figure 2, related to Figure 2 - Functional validation of the incompatible gene pair. A. Deletion of *cox15*^{stop} in the YJM421 background results in repiratory deficiency. Strains with or without deletion of *cox15*^{stop} (YJM421 *ura3Δ0 cox15*^{stop}*::URA3* and YJM421 *ura3Δ0*, respectively) in exponetial growth phase were spotted in 3 dilutions onto YPD (upper plot) and YP glycerol 2% (lower plot). Cells were grown for 48 hours at 30°C. **B.** Rescue of respiration capacity in non-viable segregants on YP glycerol 2% with *SUP7*. Respiration deficient F1 segregant was transformed with plasmids containing *SUP7* or empty control and grown for 48 hours at 30°C on YPD (upper plot) and YP glycerol 2% (lower plot).

Supplementary Figure 3.

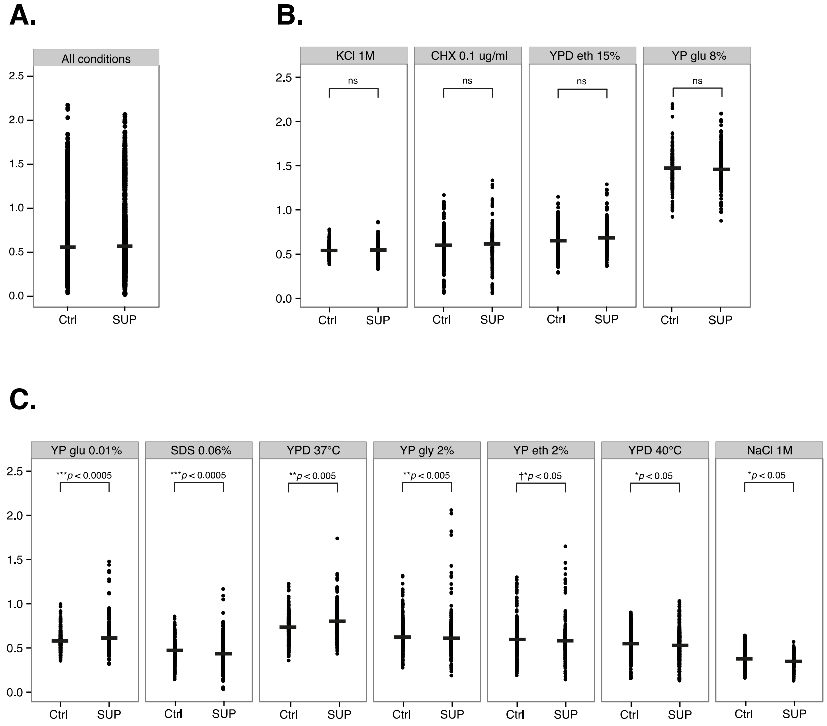

Supplementary Figure 3, related to Figure 3 - Phenotypic distribution of normalized growth ratio across natural populations in stress conditions. A. All conditions tested. Median values are indicated with a bar. Statistical tests were performed using F-test for cases with normal distribution and Levene test for multimodal distributions. **B.** Conditions with no significant change of variance. Two-sided F-test *p*-value > 0.05; Levene test *p*-value > 0.1. **C.** Conditions with significant change of variance. Two-sided F-test **p*-value < 0.05, ***p*-value < 0.005, ****p*-value < 0.0005; Levene test †*p*-value < 0.05; N=288.

Supplementary Table 1.

Strains	Source	Location	Offspring viability	Estimated divergence to S288c (%)	Experiment	Reference
**CECT10109**	Prickly pear	Spain	97%	0.31	Screen & Stress tolerance	[1]
**CLIB192**	Baker	France	92%	0.11	Screen	[1]
**CLIB219**	Wine	Russia	92%	0.44	Screen & Stress tolerance	[1]
**CLIB272**	Beer	US	95%	0.23	Screen & Stress tolerance	[1]
**CLIB294**	Fermentatio n	France	94%	0.25	Screen & Stress tolerance	[1]
**CLIB382**	Beer	Japan	92%	0.25	Screen & Stress tolerance	[1]
**DBVPG3591**	Cocoa beans		93%	0.23	Screen & Stress tolerance	[1]
**DBVPG6861**	Polluted water		96%		Screen	[1]
**EM93**	Rotting fig	US	91%	0.14	Screen	[1]
**I14**	Vineyard	Italy	94%	0.25	Screen & Stress tolerance	[1]
**K12**	Sake	Japan	91%	0.25	Screen	[1]
**PW5**	Palm wine	Nigeria	91%	0.59	Screen	[1]
**T7**	Oak tree	US	100%	0.49	Screen & Stress tolerance	[1]
**UC8**	Wine	South Africa	99%	0.28	Screen & Stress tolerance	[1]
**Y12**	Palm wine	Africa	92%	0.35	Screen & Stress tolerance	[1]
**Y3**	Palm wine	Africa	91%	0.38	Screen & Stress tolerance	[1]
**Y9**	Ragi	Indonesia	92%	0.34	Screen & Stress tolerance	[1]
**YJM269**	Apple juice		92%	0.38	Screen & Stress tolerance	[1]
**YJM280**	Clinical	US	92%	0.35	Screen	[1]
**YJM320**	Clinical	US	96%	0.32	Screen & Stress tolerance	[1]
**YJM326**	Clinical	US	91%	0.32	Screen & Stress tolerance	[1]
**YJM421**	Clinical	US	95%	0.35	Screen & Stress tolerance	[1]
**YJM440**	Clinical	US	90%		Screen & Stress tolerance	[1]
**YJM653**	Clinical	US	94%	0.32	Screen & Stress tolerance	[1]
**YJM678**	Clinical		95%		Screen & Stress tolerance	[1]
**YPS1000**	Oak exudate	US	90%	0.41	Screen & Stress tolerance	[1]

Supplementary Table 1 - Information of natural isolates used in this study

The offspring viability was estimated by dissection of 30 tetrads on YPD.

Supplementary Table 2.

Condition	Composition	Stress type	Experiment
**YPD 30°C**	1% yeast extract; 2% peptone; 2% glucose	Rich media	Screen & Stress tolerance
**YPD 37°C**	1% yeast extract; 2% peptone; 2% glucose	Rich media	Stress tolerance
**YPD 40°C**	1% yeast extract; 2% peptone; 2% glucose	Rich media	Screen & Stress tolerance
**YNB**	0.67% yeast nitrogen base w/ ammonium sulfate	Minimum media	Screen
**YP gal 2%**	1% yeast extract; 2% peptone; 2% galactose	Carbon utilization	Screen
**YP fru 2%**	1% yeast extract; 2% peptone; 2% fructose	Carbon utilization	Screen
**YP man 2%**	1% yeast extract; 2% peptone; 2% mannose	Carbon utilization	Screen
**YP sorb 2%**	1% yeast extract; 2% peptone; 2% sorbitol	Carbon utilization	Screen
**YP gly 2%**	1% yeast extract; 2% peptone; 2% glycerol	Carbon utilization	Screen & Stress tolerance
**YP eth 2%**	1% yeast extract; 2% peptone; 2% ethanol	Carbon utilization	Screen & Stress tolerance
**YP glu 8%**	1% yeast extract; 2% peptone; 8% glucose	Carbon utilization	Stress tolerance
**YP glu 0.01%**	1% yeast extract; 2% peptone; 0.01% glucose	Carbon utilization	Stress tolerance
**NaCl 1 M**	YPD; NaCl 1 M	Signal transduction	Screen & Stress tolerance
**NaCl 1.5 M**	YPD; NaCl 1.5 M	Signal transduction	Screen
**KCl 1M**	YPD; KCl 1 M	Osmotic stress	Stress tolerance
**SDS 0.03%**	YPD; SDS 0.03%	Membrane stability	Screen
**SDS 0.06%**	YPD; SDS 0.06%	Membrane stability	Screen & Stress tolerance
**CHX 0.1 µg/ml**	YPD; Cycloheximide 0.1 µg/ml	Translational stress	Screen & Stress tolerance
**CHX 0.2 µg/ml**	YPD; Cycloheximide 0.2 µg/ml	Translational stress	Screen
**YPD eth 5%**	YPD; ethanol 5%	Proteome stability	Screen
**YPD eth 10%**	YPD; ethanol 10%	Proteome stability	Screen
**YPD eth 15%**	YPD; ethanol 15%	Proteome stability	Stress tolerance
**DMSO 2%**	YPD; DMSO 2%	Oxidative stress	Screen
**DMSO 8%**	YPD; DMSO 8%	Oxidative stress	Screen
**DTT 1.5 mM**	YPD; DTT 1.5 mM	Reductive stress	Screen
**DTT 2 mM**	YPD; DTT 2 mM	Reductive stress	Screen

Supplementary Table 2, related to Figure 1 and Figure 3 - List of media conditions used and their compositions

Supplementary Table 3.

GO Biological Process (2062 categories)

Category	p-value	In Category from Cluster	k	f
**transmembrane transport [GO:0055085]**	2.76E-09	SEO1 VBA2 SUL1 PCA1 PHO89 GEX1 ERS1 GIT1 YDL199C VBA4 CCC2 NHX1 CAN1 HXT13 HVG1 FCY21 ALR2 AGP3 MAL11 DUR3 VMR1 YHK8 QDR1 DAL4 PAM16 TRK1 HXT8 HXT9 MCH2 GAP1 NFT1 GEX2 MMP1 NHA1 YLR152C AQR1 TIM23 BIO5 TAT2 HXT11 YOL162W YOL163W NRT1 THI72 COT1 SSU1 PXA1 YPR011C OPT2	49	303
**cellular monovalent inorganic cation homeostasis [GO:0030004]**	0.00029	NHX1 NHA1 VHS3	3	3
**amino acid transport [GO:0006865]**	0.0003189	VBA2 VBA4 CAN1 AUA1 AGP3 AVT3 GAP1 MMP1 BIO5 TAT2	10	42
**maltose metabolic process [GO:0000023]**	0.000415	MAL33 MAL32 MAL13 MAL11 MAL12	5	11
**cellular cell wall organization [GO:0007047]**	0.001996	ECM8 ECM11 HLR1 ECM34 ECM12 ECM14 PGU1 ECM4 CTS1 ECM19 ECM30 WSC2 TIR4 SPR1	14	89
**flocculation [GO:0000128]**	0.002621	FLO1 MUC1 FLO10	3	5
**nucleobase transport [GO:0015851]**	0.002908	FCY21 DAL4 NRT1 THI72	4	10
**amino acid transmembrane transport [GO:0003333]**	0.003968	CAN1 AGP3 GAP1 MMP1 BIO5 TAT2	6	24
**nucleobase, nucleoside, nucleotide and nucleic acid transport [GO:0015931]**	0.004332	FCY21 DAL4 NRT1 THI72	4	11
**maltose catabolic process [GO:0000025]**	0.004391	MAL32 MAL12	2	2
**glutathione transmembrane transport [GO:0034775]**	0.004391	GEX1 GEX2	2	2
**copper ion export [GO:0060003]**	0.004391	PCA1 CCC2	2	2
**cinnamic acid catabolic process [GO:0046281]**	0.004391	PAD1 FDC1	2	2
**triglyceride mobilization [GO:0006642]**	0.004391	TGL4 TGL5	2	2
**pseudohyphal growth [GO:0007124]**	0.00887	CDC39 TMN2 STE12 FKH1 MUC1 PGU1 PHD1 SPH1 DFG16 HMS1	10	64

MIPS Functional Classification (459 categories)

Category	p-value	In Category from Cluster	k	f
**amino acid/amino acid derivatives transport [20.01.07]**	0.0001246	VBA2 ERS1 CAN1 AUA1 AGP3 AVT3 GAP1 MMP1 BIO5 TAT2 NRT1	11	45
**transcription repression [11.02.03.04.03]**	0.0003351	CRF1 MIG3 RME1 HYM1 MOT3 WHI5 SFL1 ROX1	8	28
**detoxification [32.07]**	0.002122	GEX1 GRX3 VBA4 PAD1 ALR2 ROG3 SLH1 YHK8 YJR015W NFT1 GEX2 AQR1 SSU1	13	80
**transport facilities [20.03]**	0.004513	SEO1 GEX1 MCH2 PTR2 GEX2 TIM23 BIO5 YOL162W YOL163W NRT1 THI72 YPR011C OPT2	13	87
**cellular import [20.09.18]**	0.006049	APL3 SUL1 CAN1 HXT13 ALR2 DAL4 TRK1 HXT8 HXT9 FRE2 GAP1 TAT2 HXT11	13	90
**secondary metabolism [01.20]**	0.006162	COQ4 GRE3 AYT1 GCY1	4	12
**proton driven antiporter [20.03.02.03.01]**	0.008296	VBA2 ERS1 ATO2		

Supplementary Table 3, related to Figure 4 - GO term enrichment results of genes carrying TAA nonsense mutation in 100 diverse natural isolates with publically available genomes

Supplementary reference

1. Hou, J., Friedrich, A., de Montigny, J. & Schacherer, J. Chromosomal rearrangements as a major mechanism in the onset of reproductive isolation in Saccharomyces cerevisiae. *Curr Biol* **24**, 1153-9 (2014).
